# Fintech platforms: Lax or careful borrowers’ screening?

**DOI:** 10.1186/s40854-021-00272-y

**Published:** 2021-07-13

**Authors:** Serena Gallo

**Affiliations:** 1 University of Campania Luigi Vanvitelli, Capua, Italy; 2 University of Naples Parthenope, Naples, Italy

**Keywords:** Peer to peer lending, Credit grade, Misreporting, Misconduct, Recovery rate

## Abstract

Can peer-to-peer lending platforms mitigate fraudulent behaviors? Or have lending players been acting similar to free-riders? This paper constructs a new proxy to investigate lending platform misconduct and compares the FICO score and the LendingClub credit grade. To examine whether the lack of verification by the Fintech platform affects lenders’ collection performance, I explore the recovery rate (RR) of non-performing loans through a mixed-continuous model. The regression results show that the degree of prudence taken by the lending platform in the pre-screening activity negatively affects the detection of some misreporting borrowers. I also find that the Fintech platform’s missing verification information (e.g., annual income and employment length) affects the RR of non-performing loans, thereby hampering lenders’ collection performance.

## Introduction

This paper examines Fintech marketplaces’ role^1^ in affecting the credit quality of and detecting fraudulent behavior by borrowers. Lending marketplaces, also referred to as peer-to-peer (or P2P) lending, have become abundant by gaining huge market shares in consumer and small business loans over the last decade. P2P platforms are designed as a two-sided marketplace that, through leveraging innovative technologies, enables investors to lend to borrowers directly and provide broad benefits in cost and speed investment decisions. However, some suspect that the reliability of the P2P lending market has decreased over the last few years.^2^ In 2016, the Department of Justice (DOJ)^3^ and Securities Exchange Commission (SEC) accused LendingClub of false statements to financial institutions, wire fraud, and covered conduct. Renaud Laplanche was removed as CEO of the company by the board of directors. All the fraudulent activities were aimed at increasing LendingClub’s volume of loan originations by approving borrowers who did not satisfy the Credit Policy.^4^ Lending platforms have generated internal risk rating models to gauge the riskiness of underlying applicants by using sophisticated algorithms and also by relying on self-reported borrower information (e.g., annual income and employment length). With such models, lending platforms prescreen loans, list some on their websites, and allocate applications into respective risk baskets. However, traditional concerns related to the burden of information asymmetries are intensified in the unsecured online market, as economic agents have no face-face contact. Some borrowers may have incentives to alter the submitted data by inflating asset information (Lucas 1976; Jagtiani and Lemieux 2018). However, the P2P lending platform acts without skin in the game, and lenders bear the credit risk. Borrowers could be encouraged to boost loan volumes by increasing their remuneration. This work aims to analyze players’ incentives in the growing crowdfunding market. Specifically, this is a pioneering attempt to investigate how the platforms’ incentives shape their behavior, leading them to act as dishonest brokers and not identify misleading borrowers. Therefore, this work measures the platform misconduct through two proxies. The first one is the Prudence index, created to capture the borrower screening quality on the platform. The second proxy lies in the internal verification process to identify whether a lack of checking the information reported by borrowers (annual income and employment length) could hamper lenders in their collections’ performance. The goal is achieved in two steps. To start, I construct a new index that attempts to capture the degree of prudence by the platform at the loan origination. The new index is built through the computation between the FICO score (e.g., the external rating provided from Credit Agencies Bureau) and LendingClub (LC afterward), taking the following values: 1 (low prudence, as if LC has been underestimating borrower riskiness), 2 (neutral level, the assessment of borrower riskiness is the same in both models) and 3 (high prudence, as if LC has been overestimating borrower riskiness). The Generalized Ordered Logistic (Gologit) regression is implemented because of the response variable’s features. The ratios of false statements, adopted in the financial literature of borrowers’ misreporting, are used as the main predictors of the prudence index. Following Garmaise (2015), I use the ratio of rounded reported income and loan amount. The relationship between rounded self-reported value and the delinquency rate is well documented in previous works (Eid et al. 2016; Pursiainen 2020; Talavera and Xu 2018). Polena and Regner (2018)  find that more words in the loan purpose description are associated with less creditworthy borrowers and higher default rates. Based on this work, I construct a third indicator that measures the number of words provided by the borrower in the description of loan purpose. A fourth indicator finds that the borrower inflates the length of employment to access better credit-line conditions. The empirical analysis shows that borrowers with ten years of employment are associated with a higher delinquency rate. The platform’s screening quality has been decreasing over the previous years, suggesting the implementation of more aggressive underwriting to recover lost volumes. Regression results confirm that all misreporting variables negatively impact the response variable, implying that the Fintech platform does not adjust the credit grade based on the potential borrowers’ false statements. In the second step, to evaluate whether the missing platforms’ verification process may harm lenders by not granting them minimal coverage in the case of borrowers’ default, I investigate the determinants of recovery rate (RR) on non-performing loans in P2P loans. I introduce the RR modeling of defaulted loans in the P2P lending market by using a mixed continuous-discrete model already established and applied in other studies in the mortgaged market (Chawla et al. 2016; Tanoue et al. 2017). Using a novel loan-level dataset from LendingClub between July 2007 and September 2018, I test the RR’s principal determinants on defaulted loans. Because RR distribution presents a higher concentration at a zero value, I apply a mixed continuous-discrete model based on the work byCalabrese (2014).^5^ The regression results show that loan amount and the interest rate are positive determinants of the RR; unverified loans, rating volatility, and the number of borrower delinquencies negatively impact the RR. I test the robustness of the results by implementing the regressions within each risk class, leading to similar conclusions. The relationship between unverified loans and the RR is significantly negative. Therefore, if the P2P platform had enacted the verification process on information self-reported by borrowers, the RR and the loss given default would be higher and lower, respectively, resulting in better lenders’ collection performance. As a robustness check, I regress the variable verification process on the probability of default in an unreported analysis. The results confirm a positive relationship between the verification process and the borrowers’ default. However, to first address potential endogeneity by correcting for omitted variables, I have rerun the regression analysis by including the rating grade as a predictor. The positive relationship between the verification process and the probability of default is still held, as shown in Table 12. I split the initial sample by rating classes to further strengthen the results, proceeding with regressions for the three loan risk classes (e.g., A-B, C-D, and E-F-G). The positive relationship between the verification process and the probability of default is still confirmed in all risk classes, as shown in Table 13. This finding suggests that the verification process of borrowers’ self-reported information should be improved. Thus, this result may identify some negligent and opportunistic behavior of the online lending platform. This study contributes to the literature in several ways. First, the paper contributes to the burgeoning literature on the P2P lending market, filling a literature gap by examining the trade-off between maximizing profits and inferring borrowers’ quality in a completely unbiased manner. Thus far, the literature on the online lending market has mainly focused on how borrower’ soft and hard information affects the likelihood of default (Emekter et al. 2015; Carmichael 2014; Lu et al. 2012; Serrano-Cinca et al. 2015; Polena and Regner 2018) and the roles of alternative data and machine learning in improving access to credit and screening quality (Berg et al. 2018; Balyuk and Davydenko 2019; Duarte et al. 2012; Everett 2015; Freedman and Jin 2017; Hertzberg et al. 2018; Jagtiani and Lemieux 2018; Pope and Sydnor 2011; Shen et al.  2021). To the best of my knowledge, this is the first study to investigate how the Fintech platform could affect loan screening by inflating the borrower’s quality. Iyer et al. (2016) and Vallée and Zeng (2019) study how peer lenders can predict an individual’s likelihood of defaulting on a loan with greater accuracy than the borrower credit score, showing that sophisticated investors screen loans differently. Second, this work adds to the extensive stream of research on the financial and accounting misconduct that encompasses the relationship between CEO equity incentives and false statement (Bergstresser and Philippon 2006; Burns and Kedia 2006; Cheng and Warfield 2005; Efendi et al. 2007; Jensen and Meckling 1976; Efendi et al. 2007). It is also linked to this stream of research because similar to how CEOs could have personal incentives to falsify corporate balance sheets, the P2P lending platform could underestimate the borrowers’ credit risk to increase their remunerations by not adopting due diligence (Cumming et al. 2019). Third, the study integrates the literature on borrower misreporting in the mortgage market that finds a strong association between borrower’ misreporting and adverse loan outcomes (Agarwal and Ben-David 2014; Garmaise 2015; Griffin and Maturana 2015; Jiang et al. 2014; Piskorski et al. 2015). Also, this study is linked to works by Oleksandr and Xu (2018) based on loan verification and Pursainen (2020) that show that the LendingClub platform does not adjust the pricing on loans for misreporting borrowers. Finally, this paper contributes significantly to the growing literature on the estimation of the loss given default (LGD) and RR in the unsecured market (Calabrese 2014; Gourieroux and Lu 2019; Ye and Bellotti 2019; Siao et al. 2016; Zhou et al. 2018), advising lenders to focus on additional credit risk measures to accurately assess borrower creditworthy. In marketplace lending, the information asymmetries between borrowers and lenders lead to higher default rates and large LGD. Additionally, this study provides valuable insights to policymakers by highlighting critical factors that could lead to financial stability concerns due to the drying up of funding due to consumers’ loss of confidence. The paper also gives novel practical insights for lending platforms that might represent a concrete solution to credit rationing. Through its results, this study provides suggestions for lending platforms to improve the loan verification process to detect misreporting information by some borrowers and to strengthen their internal corporate governance, for instance, through the adoption of measures aimed to punish false statements by some applicants. The remainder of the paper is organized as follows. Related literature is reviewed in second section. Summary statistics are reported in third section. The empirical methodologies on the RR and prudence index are in fourth and fifth sections, respectively. Sixth section concludes the manuscript.

## Related literature

### Financial misreporting fraud

The extensive literature on financial misreporting fraud has examined why managers engage in corporate earnings by analyzing the equity incentives to misreport (Bergstresser and Philippon 2006; Burns and Kedia 2006; Cheng and Warfield 2005; Efendi et al. 2007; Jensen and Meckling 1976).^6^ Misconduct, however, is an inevitable effect of the capital market. The burden of the analyst’s forecasts would bring pressure on managers, who are willing to destroy the value of firms to avoid severe punishment ofhe market (Degeorge et al. 1999). Financial misreporting may be facilitated when the CEO is also the firm’s founder, serves as chairman, or belongs to the founding family members^7^ (Agrawal and Chadha 2005; Dechow et al. 1996) because of more reliable connections with other top executives and directors (Altunabas et al. 2018; Khanna et al. 2015). The economic literature is rich, with empirical and theoretical studies highlighting the role of reputational loss in deterring financial misreporting and aggressive accounting policy (Giannetti and Wang 2016; Karpoff and Lott 1993; Murphy et al. 2009).^8^ The monetary penalties for sued firms are lower than reputational loss imposed by the market (Karpoff and Lott 1993), which are nearly nine times the size of fines associated with wrongdoings. Thakor and Merton (2018) assert that trust is more difficult to gain than to lose. Its asymmetric nature could be enhanced in the P2P lending market because of the weaker incentives to maintain it than the traditional banking system. Banks, therefore, could have more substantial incentives to make good loans because they use the money raised through deposits, and the damage to the lender’s trust can endanger future fundraising, whereas Fintech platforms are investor-financed. The platforms’ incentives may impact the ability to distinguish between misleading and truthful borrowers. This stream of research is related to the liars’ loan problem, discussed widely in the mortgage loan market following the financial crisis (Jiang et al. 2014). Griffin and Maturana (2015) have sought to identify potential fraud through three indicators of misreporting on low and total documentation loans, finding that approximately 48% of loans had at least one sign of misrepresentation. Empirical evidence on mortgage loans shows that borrowers’ reporting asset information above the threshold rather than those just below were almost 25% points more likely to become delinquent (Garmaise 2015). On this basis are built the works by Eid et al. (2016) and Pursainien (2020) that, using a complete dataset from LendingClub, revealed that borrowers with a tendency to round their income are more likely to default than those with more accurate income reporting. Also, lenders are not compensated for additional risk associated with rounding borrowers priced with a lower interest rate. Despite their limitations, the studies mentioned above collectively explain why misconduct has become an important issue and potential proxies to measure misconduct risk in the financial market.

### P2P loan performance and lax screening

A large body of contemporary studies has examined different features of P2P lending**.** The first stream of research has focused on the importance of soft and hard information in mitigating asymmetric information in borrower-lender interactions. The traditional bankruptcy prediction models for small and medium enterprises (SMEs) use accounting-based financial ratios typically. Kou et al. (2021) have proved the economic benefit of transactional data and payment network-based variables for bankruptcy prediction. Several aspects can contribute to predicting the credit risk of borrowers,^9^ such as the economic value of networks and online friendships (Lin et al. 2013; Freedman and Jin 2017), maturity choice of loans as a signal of the higher risk of worsening of creditworthiness (Yao et al. 2019; Hertzberg et al. 2018), social media information (Iyer et al. 2016), digital footprint (Berg et al. 2018; Ge et al. 2017) and borrowers’ characteristics (Carmichael 2014; Emekter et al. 2015; Serrano-Cinca et al. 2015).^10^ According to Basel Accords, investors should be mindful of the default rates and LGD in making investment decisions and assessing credit risk for loans. Recently, studies have sought to evaluate the LGD in the P2P setting. For instance, Zhou et al. (2018) present the first model of LGD, using data from LendingClub, and describe the probability density function of LGD as a unimodal distribution with the high value peaking in the unsecured bond market. They also find negative relationships between credit grade, debt-to-income ratio, and LGD, and that borrowers’ total assets do not have a significant impact. In contrast, Papoušková and Hajek (2020) assert that LGD does not follow the normal distribution, and they have adopted a random forest learning method to reduce overfitting. I follow the perspective by Ye and Bellotii (2019) and Calabrese (2014) that have used beta mixture regression in modeling RR on non-performing loans in the mortgage market. Furthermore, the literature has mainly examined the relationship between lenders and borrowers in the P2P lending context, looking at the platform as an honest broker and borrowers as misleading users. According to Cumming et al. (2019), one important issue is what role the platform should play in the governance of crowdfunding marketplaces. Fee structures in the lending market affect how platforms carry out their core business, seeking to maximize the revenue they make. Fraser et al. (2015) state that although online platforms can disentangle financial constraints, their role in the context of monitoring and governance is still unclear. However, platforms’ activity should be examined because they serve a double purpose: they are at the same time a credit agency in screening loans and providers of investment decisions (Bertsch and Rosevinge 2019). Banks retain a fraction of all originated loans, thus acting as a signal of asset quality by ensuring that they have skin in the game^11^ (Daley et al. 2020), unlike P2P platforms that are reluctant to retain a fraction of originated loans. Likewise, the rating issue from the Credit Rating Agency (CRA), which has skin-in-the-game requirements, is more accurate than those who do not have these requirements (Ozerturck 2015). According to Lucas’s critique (1976), a statistic model could be deceiving because agents’ incentives change or alter data’s real nature.^12^ Platforms might be tempted to reduce lending standards by offering too many low-quality loans to boost loan volume beyond sustainable levels, thereby negatively affecting unskilled investors who rely on its judgment (Balyuk and Davydenko 2019). Consistent with this view, Keys et al. (2010) empirically demonstrated that the securitization process affected the adverse selection problem by increasing financial intermediaries’ incentives to screen borrowers carelessly. Recently, few studies have attempted to evaluate the effectiveness of the credit scoring systems used by P2P lending platforms. Wang et al. (2021) state that the credit rating of loans is vital in assessing default risk. Their study is the first to study cost-sensitive classifiers and measure misclassification costs of different credit grades in P2P lending. Jagtiani and Lemieux (2018) have found a lower correlation between FICO scores and LC grade from approximately 80 to 35% for loans that originated in 2014–2015. They state that a significant portion of borrowers, previously classified as subprime based on the FICO score, are slotted into a better risk class. Gao et al. (2017), using the loan data from Renredai.com, a Chinese P2P lending platform, have classified the platform’s evaluation systems as forward-looking based on borrowers’ information, with backward-looking mechanisms based on their historical repayments. They have shown that the backward-looking system encourages bad borrowers to default after they have earned high enough credit scores to borrow a large amount, suggesting the need to improve the credit scoring model. Talavera et al. (2018), using data from a leading Chinese lending platform, prove a positive relationship between the default rates of loans and borrowers with incomplete verified information. The LendingClub platform asks borrowers to provide some personal information. Specifically, the self-reported data are annual income and length of employment. LendingClub could ask the potential borrower to verify the self-reported information or only its source, for instance, the source of income or the company where the borrower works. Some borrowers obtain funding without information verification. Therefore, the verification process seems to be a subsidiary activity in the lending market (Carmichael 2014; Jagtiani and Lemieux 2018; Polena and Regner 2018). The adoption of due diligence mitigates potential reputation costs and litigation resulting from loans that should not have been originated due to lower quality (Cumming et al. 2019). Tao et al. (2017) also note that because of the lack of official credit records of borrowers and the information submitted by themselves on which the platforms’ credit rating system is based, inaccurate or false data is not easily identifiable in the verification process. Platforms should use due diligence in adopting a more robust verification mechanism to improve the efficiency of the crowdfunding market. Therefore, the verification system could offer an alternative way to decrease adverse selection problems, not only in detecting fraud and liars’ loans by validating borrowers’ documentation but also, according to Signaling Theory (Spence 1973), as a signal of the asset quality by increasing its reputation. For instance, Renredai.com has developed both online and offline verification tools, such as physical site visits, to check the information submitted by borrowers, increasing lenders’ trustworthiness and guaranteeing the survival of the crowdfunding market (Huang et al. 2021; Tao et al. 2017).

### The hypothesis development

According to prior studies that have used different measures and explanations to explore how players’ incentives affect their conduct in the market (Chami et al. 2010; Gorton and Pennacchi 1995; Mason et al. 2009), more research is necessary to understand this issue in the crowdfunding market. Hildebrand et al. (2017) examined the players’ incentives in the crowdfunding market for the first time by providing empirical evidence on adverse incentives that are not fully recognized in the market. However, only a few studies have attempted to explore the role of online lending platforms in assessing and monitoring borrowers’ creditworthiness with conceptual discussion. The crowdfunding platform is driven by profit and ethical or reputational concerns (Cumming et al. 2019; Hildebrand et al. 2017). The impact of fee structures on their behaviors in the crowdfunding market remains unclear. To fill this gap, in this paper, I investigate the linkage between the lending platform’s prudence degree, the proper detecting of misleading borrowers, and the lenders’ collections performance. From the discussions above, I have drawn the following hypothesis:

#### H_1_

P2P lending platforms’ incentives affect the signaling of misreporting borrowers, thereby hampering lenders’ collection performance (e.g., recovery rate).

**Fig. 1:**
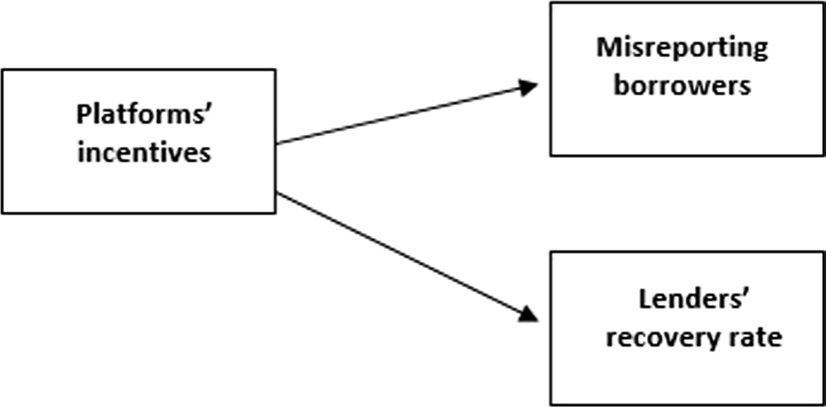
Theoretical framework

The theoretical framework is shown in Fig. 1.

To test this hypothesis, I construct a new proxy of platforms’ misconduct by comparing the LC rating grade and FICO score. I adopt this index to explore whether the assessment of borrowers’ riskiness through automated credit grading algorithms based on machine learning techniques (e.g., LC rating grade) is different from traditional credit scoring (e.g., FICO score). Therefore, if the platform has been underestimating borrowers’ riskiness, it could harm lenders’ collections in the case of borrowers’ default. The platforms’ incentives have significant implications for both lenders and borrowers because improper conduct of platforms may lead to the collapse of the crowdfunding market (Hildebrand et al. 2017; Vismara 2018).

## Data description and descriptive statics

### LendingClub operativity overview

The research is focused on LendingClub, which is a leading platform in the US that was established in 2007; it was the first lender to register its offerings as securities with the SEC. By the end of December 2019, it had provided almost 3 million loans, with a total lending amount of over $42.53 billion. Borrowers need to comply with the requirements of the platform to successfully apply for a loan. For instance, LC rejects borrowers with FICO scores less than 600, a credit history of less than three years, and a debt-to-income ratio of more than 40%. The potential borrowers have to report their annual income, employment status, current home situation, and other personal information. If they overcome the constraints, LC assigns them a fixed interest rate based on its credit grades, which range from A (the lowest risk) to G (the highest risk), with subgrades from A1 to G5. For instance, the average interest rate for A1 was 5.53% and for G5 was 29.14% between July 2007 and September 2018. Typically, Fintech platforms might verify if their reported income is within 10% of their actual income or the employment source. However, the platforms grant loans before conducting the verification because it has made only a target of loans. If the borrowers’ self-reported data are not truthful, such as overstated income, their applications cannot be removed, and they can still go ahead with loans without any penalty. Furthermore, LC has tested a new verification process in the fourth quarter of 2018 to reduce friction for borrowers seeking loans. Consistently, LC does not guarantee the trustworthiness of borrowers’ data, but it attempts to take reasonable actions in mitigating liars’ applications.

### Sample construction and descriptive statistics

As outlined in the previous section, I use a personal loan dataset from LendingClub, which encompasses all consumer loans issued between July 2007 and September 2018. The sample ends in the third quarter of 2018 to observe loan performance over almost 2 years post-origination. I drop loans that did not meet the credit policy, which originated between 2009 and 2010.^13^ The total sample contains 1,959,440 loans. The detailed descriptions and the descriptive statistics of the variables used in the empirical analyses are reported in Table 1. In constructing the prudence index and misreporting variables, I focus on consumer loans, both current and mature. For the RR and LGD analysis, the empirical study only involves mature loans, resulting in 1,494,741 borrowers’ records.

**Table 1:** Variable description and descriptive statistics

Variable	Description	Obs	Median	Average	SD	Plst	P99th
Loan amount	Loan amount chosen by borrower	1,959,405	13,000	15,109	9,161	1,600	40,000
Loan amount^2^	Squared term of loan amount	1,959,405	89.73	89.10	12.79	54.43	112.28
Annual income	Borrower’s stated annual income	1,959,332	69,959	79,563	116,697	20,000	275,000
DTI	Debt to income ratio of loan applicant	1,954,693	17.69	18.50	11.46	1.76	39.89
Delinquencies 2 years	The number of 30 + days past-due delinquency in the last 2 years	1,954,784	0	0.31	0.88	0	4
Account open	Number of trades open	1,954,784	11	11.69	5.63	3	30
Total account	Number of total account	1,954,784	23	24.34	11.99	5	60
Employment length	Employment length in years at listing of loan request	1,959,332	3	4.56	3.17	1	11
Inquires last 6 mths	The number of borrower’s inquires in the last 6 months	1,954,783	0	0.58	0.88	0	4
LendingClub grade	Internal rating assigned by the platform. The grade can take values from A = 7 (the lowest-risk class) to G = 1 (the highest-risk class)	1,959,405	5	5.33	1.26	2	7
Fico score	External rating assigned by Credit Agency Bureau. The rating takes different classes from the lowest to highest risk: 1) < 660; 2)660–679; 3) 680–699; 4) 700–739; 5) 740–759; 6)760–779; 7) 780 +	1,954,784	692	700	32.62	662	802
Not verified	Dummy variable. It takes value 1 if borrower’s stated information is not verified, 0 otherwise	1,959,332	0	0.32	0.47	0	1
Default	Dummy variable. It takes value 1 if loan is defaulted or charged-off within 12–24 months post-duration, 0 otherwise	1,494,741	0.19	0.39	0	0	1
Interest rate	The loan interest rate sets by LendingClub	1,959,405	0.12	0.13	0.04	0.05	0.27
Term 5 years	Dummy taking the value 1 if the loan’ term is 5 years, 0 otherwise	1,959,405	1	1.27	0.44	1	0
Income divisible by 5000	Dummy taking the value 1 if the borrower'reported income is divisible by 5,000, 0 otherwise	1,959,332	0	0.49	0.5	0	1
Income divisible by 10,000	Dummy taking the value 1 if the borrower’reported income is divisible by 10,000, 0 otherwise	1,959,332	0	0.29	0.45	0	1
Loan amount divisible by 5000	Dummy taking the value 1 if the loan amount is divisible by 5,000, 0 otherwise	1,959,405	0	0.33	0.47	0	1
Loan amount divisible by 10,000	Dummy taking the value 1 if the loan amount is divisible by 10,000, 0 otherwise	1,959,405	0	0.17	0.38	0	1
Revolving utilitation	The revolving credit utilitation of the borrower	1,953,322	0.50	0.50	0.24	0.01	0.98
Home owned	Dummy taking the value 1 if the Homeownership status provide by borrower is “own”, 0 otherwise	1,959,322	0	0.10	0.30	0	1
Home mortgaged	Dummy taking the value 1 if the mortgaged status provide by borrower is “mortgage”, 0 otherwise	1,959,322	0	0.49	0.5	0	1
Prudence grade	Prudence of the screening system. It is calculated as the difference between LendingClub grade and Fico score. It takes value 1 (Low Prudence), 2 (Medium) or 3 (High Prudence)	1,959,405	1	1.18	0.48	1	3
Recovery rate	Recoveries amount on defaulted loans	291,665	0.088	0.098	0.11	0	0.45
Year	Dummy taking the value 1 if the loan is issued between 2016 and 2018, 0 otherwise	1,325,744	1	0.57	0.49	0	1
Suspect employment length	Dummy taking the value 1 if the length of employment is equal at 10 years, zero if it is less 10	1,959,440	0	0.40	0.49	0	1
Length of title	Number of words provided by borrower in the title of loan	1,959,440	2	2.147	0.69	0	18
Amount to income	Amount of the loan issued to the annual income provided by borrower	1,959,250	0.2	0.31	51.09	0.02	0

This table reports the description and the main descriptive statistics the variables involved in the empirical analysis. The sample covers all loans issued from LendingClub between July 2007 and September 2018. The point and comma are used as decimal and thousand separators. The construction of the variables Prudence grade and Recovery rate is presented in the following sections

**Fig. 2:**
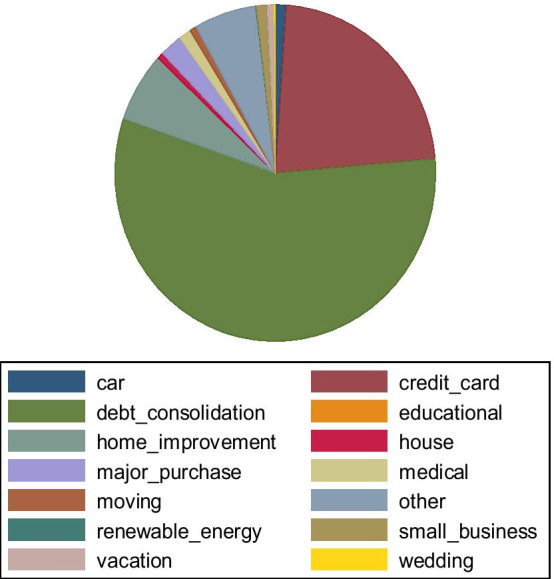
Consumer loans by the stated purpose. Notes. This graph shows loan distribution by a self-reported goal by borrowers. As highlighted, a large part is specified to be used for consolidating borrowers’ liabilities

As shown in the table above, the average loan amount selected by borrowers is approximately $15,000, and the average interest rate of the loan is 13%. LendingClub provides two types of loans: 36-month short-term loans and 60-month long-term loans. In the sample, 60% of loans have a short maturity of approximately five years. For borrowers’ self-reported data, the average annual income is $79,563, with approximately 5 years of employment. Almost 50% of applicants have a mortgaged home, and only 10% own one. On applicants’ indebtedness features from the Credit Bureau, most applicants have low FICO scores of approximately 700. However, the loan applicants have an average of 12 credit lines opened and approximately 24 completed lines, and the median rate of utilization is approximately 50%. Concerning the verification of reported data by borrowers, it emerges that 32% have neither stated income nor verified their employment status; in contrast, another 40% are source verified, and the remainder are both. Approximately, 78% of consumer loans are represented by credit card and debit consolidation purposes, as shown in Fig. 2. The distribution of loans by status in the sampling period is shown in Table 2.

**Table 2:** Loan status by each year

	Number of loans by Status						
	Charged off	Current	Default	Fully paid	In grace period	Late (16–30)	Late (31–120)
2007	38			175			
2008	124			689			
2009	406			2882			
2010	1131			7092			
2011	2351			12,826			
2012	6,254			32,025			
2013	14,895			79,938			
2014	28,471	2685		128,360	48	23	110
2015	55,321		3	217,810			
2016	44,662	41,497	2	172,906	681	294	1804
2017	39,797	128,248	98	131,319	2169	867	4025
2018	19,813	159,716	152	64,961	2581	825	4093
Total	213,263	332,146	255	850,983	5479	2009	10,032

This table provides summary statistics on the data set used for the empirical analysis. The data covers all loans issued from LendingClub between July 2007 and September 2018. The status of loan by each year is reported in the table. According to LendingClub, a loan becomes "Default" when borrowers miss payments for an extended period. Charged off is a loan for which is no longer a reasonable expectation of further payments, and it occurs when borrowers are 120 days or more past due

### Evaluation of prescreening activity

**Fig. 3:**
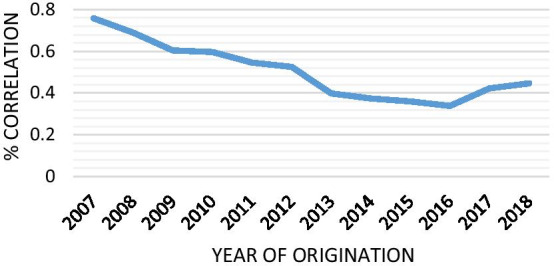
Correlation between FICO score and the LC rating grade. Notes. This graph displays the association between two rating systems on the whole data set, involving also current loans. As can be seen, their relationship has been changing over time, confirming a declining trend. Nevertheless, the relation in 2016 seems to improve slightly once again

**Fig. 4:**
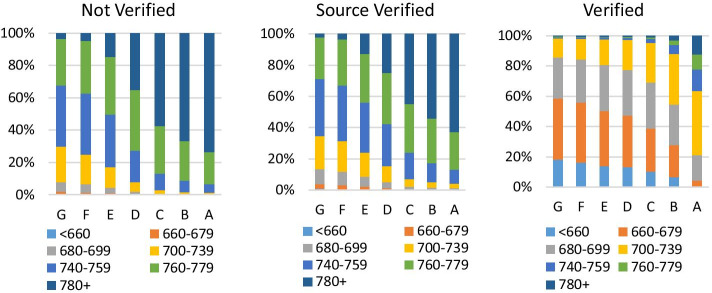
Fico and LC grade distribution by Verification status. Notes. These graphs show the relationship between FICO and LC grade by loans’ verification status. The empirical analysis is being performed on the whole data set, including loans issued between 2007 and Q3 2018

The weakness of the control system could incentivize some borrowers to inflate or falsify their self-reported information. These sections investigate the prescreening quality at the time of loan origination, using as a proxy of platform misconduct the degree of prudence adopted by the platform in allocating borrowers in specific risk classes. The empirical analysis is performed with loan-level data from LendingClub, including current loans issued between July 2007 and September 2018. Firstly, I begin by evaluating the FICO score and LC grade discrepancies through the correlation analysis. As shown in Fig. 3, the relationship has decreased from 76% for loans issued in 2007 to 40% in 2018. Figure 3 presents the composition of loans for each LC rating grade and FICO score based on the loan verification status. Some consumers, defined as subprime with a FICO score below 680, are slotted into better loan classes based on LC’s rating grade in the unverified and source verified stage (Fig. 4).

**Fig. 5:**
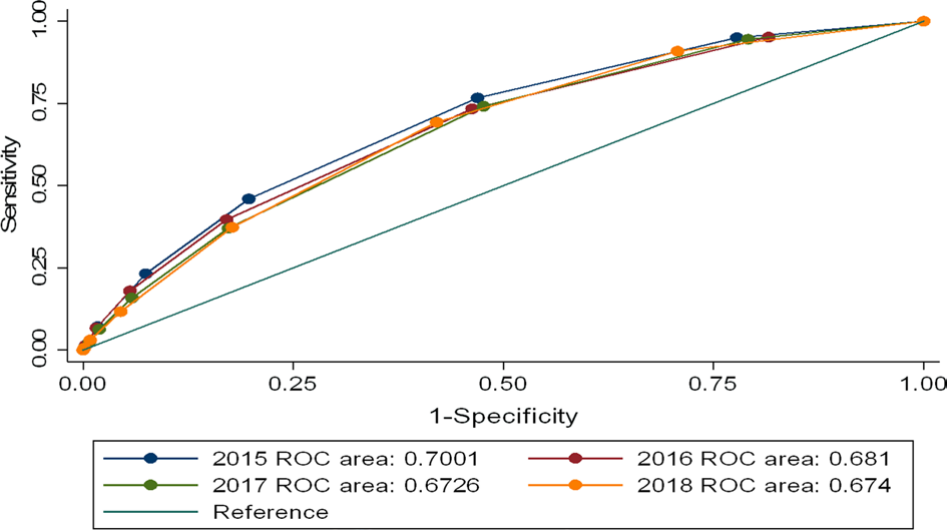
ROC curve of Lending Club grades. Notes. This Figure plots the ROC curve, which plots the positive true rate, also called Sensitivity versus true false rate (1-Specificity), obtained by using the LC rating grade as a predictor of defaulted loans. The analysis is performed each year separately on loans issued between 2016 and 2018. The larger the AUC, the better the model is

Consequently, I focus on the ability of the LC rating grade to infer borrower quality to evaluate the prudence grade of the LendingClub marketplace over time. A higher prescreening activity means good loans are more accurately distinguished from bad loans, screened out, or in a risk bucket. Following the literature (see Iyer et al. 2016; Vallèe and Zang 2019), I measure the accuracy of the LC prescreening activity by building receiver operating characteristic (ROC) curves. To perform our analysis, I assess the likelihood of charged-off loans using LC class grade as predictors. Thus, a higher AUC means that the system is a good predictor of defaulted loans.^14^ The test is computed separately on loans between 2015 and 2018 to capture how the screening quality of Fintech platforms evolves. ROC analysis results are displayed in Fig. 5, showing that the predicted power for defaulted loans of LC’s rating grade has decreased by 0.03 percentage points over the last years.

## Prudence index

To proxy the degree of prudence of the platform’s risk management, I construct a prudence index, defined as the difference between LC credit grade and FICO scores, namely internal and external ratings, respectively. The prudence index attempts to capture the underestimation of risk by Fintech platforms. I aim to investigate the determinants that affect prudence taking by the lending platform. They might be encouraged to excessively underestimate credit risk to increase their remuneration. Firstly, I operationalize LC’s rating grade from categorical to continuous, where G is 1 (Highest risk), F is 2, … and A is 7 (lowest risk). For instance, if the LC rating grade assigns borrowers into the G risk class, this means that they have a higher likelihood of defaulting on loans. Then, we classify FICO scores into seven segments^15^ from the lowest to highest score, in which borrowers with high credit risk are assigned to the lowest score, for instance, a score lower than 660. The splitting of the FICO scores within different risk buckets is based on the work by Balyuk and Davydenko (2019). The prudence index is built through the difference between the two reclassified credit risk systems by taking three levels: low prudence = 1, same prudence = 2, and high prudence = 3. For instance, the difference between LC and FICO scores is greater than 1 when a borrower receives a score equal to 3 by LC and equal to 1 by FICO. This means that LC allocates some borrowers in a lower risk class (i.e., 3) than FICO, which assigns the same borrowers in a higher risk class (i.e., 1). In the opposite case, if the difference is lower than 1, LC allocates some borrowers to a higher risk class (i.e., 3), while FICO assigns them to a lower risk class (i.e., 1).

**Fig. 6:**
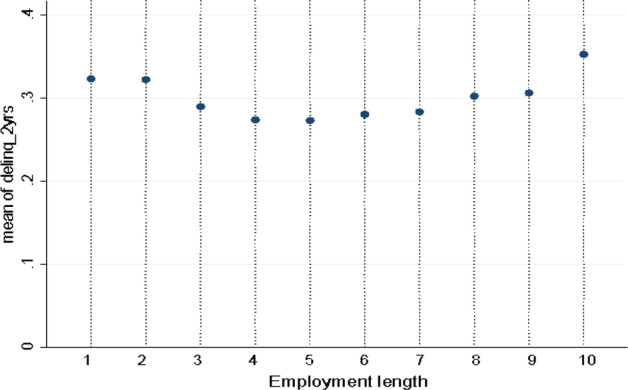
Employment length by delinquency rate. Notes. This graph shows the employment length by the average delinquency rate. The empirical analysis is performed on the loan-level dataset issued from LendingClub between June 2007 and September 2018. Both current and matured loans are included. The maximum level of the working year is associated with the highest rate of delinquency

In this case, the FICO score has underestimated the borrower’s riskiness. Instead, both FICO and LC assess borrowers’ riskiness in the same way in the middle case, for instance, when the two credit rating systems assign the same score to borrowers. The index takes value 1 when the LC rating grade underestimates the borrower’s risk (lowest prudence); 2 if the LC credit assessment is similar to FICO, and 3 when the LC rating is more prudent than the FICO score (highest prudence). In the empirical analysis, the prudence index takes value 1 for approximately 88% of the whole sample, confirming that LC’s rating grade has included borrowers as A-rated or B-rated most of the time. Therefore, to assess the LC marketplace’s screening prudence, we use misreporting variables that have been already used in literature and are associated with significantly higher borrowers’ delinquency rates. Following previous studies on the financial literature, the borrower’s inaccurate or untruthful information can signal potential misreporting. Following behavioral studies, when people are asked to estimate a value, they are inclined to provide a rounded estimation. This tendency is more likely in people lacking specific knowledge or documentation. Previous studies on this topic have also shown that borrowers reporting above-rounded number values for their assets have significantly higher delinquency rates in the P2P lending market (Eid et al. 2016). P2P loans with a goal amount to a round number are associated with a lower probability of finding success in the reward-based crowdfunding market (Lin and Pursiainen 2018). Based on potential misreporting indicators established in the financial literature (Garmaise 2015; Pursiainen 2020), I identify the self-reported annual income of borrowers as misreporting when it is divisible by 5,000 and 10,000. The cut-off points are set in the literature and reflect the rounding of values reported by people. Based on previous works by Garmaise (2015), Eid et al. (2016), and Pursiainen (2020), we adopt the following misreporting indicators taking true value as when 1) reported income is divisible by 5,000 and 2) reported income is divisible by 10,000, 3) the loan amount is divisible by 5,000, and 4) the loan amount is divisible by 10,000. I add to the literature on loans with the following variable: 5) the length of loan title provided by borrowers and 6) the suspicion of a false statement about the length of employment. Table 3 lists summary statistics for misreporting variables, and in Fig. 6, the relationships between the delinquency rate and the length of employment at the maximum level are displayed.

**Table 3:** Summary statistics of misreporting variables

Variables	Obs	Mean	SD	Min	Max	p25	p50	p75
Prudence grade	1,959,405	1.182	0.485	1	3	1	1	1
Income divisible by 5000	1,959,332	0.296	0.457	0	1	0	0	1
Income divisible by 10,000	1,959,332	0.497	0.5	0	1	0	0	1
Loan amount divisible by 5000	1,959,405	0.337	0.473	0	1	0	0	1
Loan amount divisible by 10,000	1,959,405	0.179	0.383	0	1	0	0	0
Length of title	1,959,440	2.148	0.691	0	18	2	2	2
Suspect employment length	1,959,440	0.403	0.491	0	1	0	0	1

This table provides summary statistics on the variables used for the empirical analysis of the LendingClub' Prudence grade. The data covers all the transactions between June 2007 and September 2018. Mean, Standard deviations, key percentiles and minimum and maximum values are displayed

### Factors affecting the degree of prudence

Thus far, the analysis results indicate that LC rating has facilitated the slotting of some borrowers into a better risk class compared to the external rating. However, this may lead to an excessive underestimation of borrower risk by destroying lenders’ profits. I use a proxy to measure screening quality and the prudence index explained in the last section. I aim to investigate this issue and the determinants that affect the degree of prudence of LendingClub. The generalized ordered logit estimated was estimated as follows (Williams 2006):

1 $$\mathrm{ln}\left({\mathrm{Y}}{^\prime}_{\mathrm{I}}\right)={\mathrm{\alpha }}_{1}+{\upbeta }_{1 }\times{{\mathrm{Rounded}}_{\mathrm{Income}}}_{\mathrm{i}}+{\upbeta }_{2 }\times{\mathrm{Rounded}\_\mathrm{Amount}}_{\mathrm{i}} +{\upbeta }_{3 }\times\mathrm{Lengt}{\mathrm{h}\_\mathrm{Title}}_{\mathrm{i}} +{\upbeta }_{4 }\times\mathrm{Suspect}\_\mathrm{emp}+ {\upbeta }_{5 }\mathrm{x }{\mathrm{X}}_{\mathrm{I}} +{\upvarepsilon }_{\mathrm{I}}$$

The dependent variable is the degree of prudence of the LC rating grade ranging from 1 to 3. Following the literature on borrower misrepresentation, the income roundness, the loan amount roundness, the number of words provided by borrowers in the title description, and the suspicion of inflating the working years are used as predictors. X_i_ is a vector of the control variable, including loan information and borrower’s characteristics. The Gologit model estimates the odds of being beyond a certain category (highest prudence) or to be at or below that category (lowest prudence). The Brant Test was used to evaluate the proportional odds (PO) assumption, resulting in the violation of some covariates. Following Williams (2006), we use the partial proportional odds model, which holds constant covariates that meet the PO and allows one or more coefficients to move freely across different categories of the response variable. For the three types of the dependent variable, two equations were fitted. Some variables have a single constant odds ratio across all the three equations because the PO is fulfilled, for instance, debt-to-income ratio, home mortgaged, length of employment, and annual income. In contrast, other variables have different coefficients for each of the prudence categories, and their effects vary across the levels of the response variable. Table 4 displays the results.

**Table 4:** Regressions results

	(1)		(2)		(3)	
	Low-prudence	Medium	Low-prudence	Medium	Low-prudence	Medium
Not verified	0.769*** (0.00433)	0.598*** (0.00626)	0.637*** (0.00349)	0.468*** (0.00479)		
Income div. 5000	0.915*** (0.00591)	0.898*** (0.00966)				
Income div.10,000	0.989*** (0.00702)	0.960*** (0.0114)				
Loan amount div. 5000			1.089*** (0.00717)	1.133*** (0.0124)		
Loan amount div. 10,000			0.791*** (0.00652)	0.718*** (0.0100)		
Length of title					0.975*** (0.00657)	0.924*** (0.0101)
Suspect empl. length					1.046*** (0.00634)	1.024*** (0.0103)
ln(Loan amount)	0.00408*** (0.000296)	0.00361*** (0.000461)			0.0053*** (0.000476)	0.00446*** (0.000686)
Loan amount^2^	1.390*** (0.00542)	1.410*** (0.00960)			1.379*** (0.00655)	1.408*** (0.0115)
Debt to income ratio^α	1.036*** (0.000300)	1.039*** (0.000483)	1.037*** (0.000309)	1.039*** (0.000501)	1.038*** (0.000372)	1.042*** (0.000601)
Months since recent inquires	0.981*** (0.000432)	0.964*** (0.000736)	0.982*** (0.000429)	0.966*** (0.000733)	0.981*** (0.000528)	0.965*** (0.000891)
Revolving utilitation	0.0753*** (0.000850)	0.0674*** (0.00115)	0.0867*** (0.000963)	0.0798*** (0.00134)	0.0935*** (0.00130)	0.0990*** (0.00208)
Account open	0.975*** (0.000771)	0.983*** (0.00120)	0.970*** (0.000768)	0.980*** (0.00120)	0.983*** (0.000947)	0.997* (0.00146)
Delinquencies in last 2 years	0.710*** (0.00305)	0.701*** (0.00477)	0.694*** (0.00298)	0.687** (0.00465)	0.707*** (0.00366)	0.704*** (0.00564)
Home mortgaged					0.983** (0.00665)	0.858*** (0.00955)
Home owned^α					1.084*** (0.0110)	1.077*** (0.0175)
Employment length^α			1.015*** (0.000743)	1.014*** (0.00124)		
Loan purpose: vacation^α					0.901*** (0.0308)	0.892*** (0.0495)
Loan purpose: credit card					0.289*** (0.00423)	0.205*** (0.00516)
Loan purpose: debt consolidation					0.525*** (0.00493)	0.449*** (0.00651)
Loan purpose: small business					1.426*** (0.0334)	1.481*** (0.0458)
Loan purpose: Home improvement					0.662*** (0.00882)	0.569*** (0.0120)
Year					0.899*** (0.00163)	0.895*** (0.00268)
3-digit zip code					Yes	Yes
Observations	1,558,546	1,558,546	1,558,546	1,558,546	1,053,512	1,053,512

This table displays odds ratios from Gologit regressions that m-1 models, where m is the number of clusters. The dependent variable is an indicator of Prudence'screening by platforms, and the highest Prudence is the reference category. Imprudent models are displayed in columns 1, 3 and 5, and neutral groups in Columns 2,4 and 6. The variables with superscript α meet the odds assumptions and are the same in all categories (e.g. debt-to-income ratio, home mortgaged, home owned). For variables violating proportional odds assumptions, refer to coefficients for responses of 2,3 vs 1 group (Low Prudence models) and category 3 vs 1, 2 in Neutral models

There are two takeaways from this analysis. First, in the overall models, the screening activity does not improve whether the loans are not verified by the platform, revealing any negligent behavior in assessing borrowers’ creditworthiness. This effect is robust in the neutral category (3 vs 1 and 2), decreasing by 40% the odds of being above a category (high prudence) versus being in that category. Second, all misreporting variables strongly predict the screening quality by the LC rating grade, suggesting that the risk associated with these characteristics is not entirely incorporated by the platform when listing loans. These predictors assume different coefficients for each category of the response variable because Odds parallel lines assumptions are violated. Variables with an odds ratio less (higher) than 1 indicate that the LendingClub scoring models underestimate (overestimate) the borrower risk. I start to focus on Model 1, in which only two misreporting variables were included. For instance, borrowers who report incomes rounded to the threshold of 5,000 are negative predictors of the dependent variables, suggesting that with one unit increase in the roundness of income, the prudence quality decreases by 8.5% and 10.2%.^16^ Consistent with our view, the variable loan amount seems to decrease the odds of prudence, suggesting an underestimation of the borrower risk again. Conversely, the squared specification of loan amount indicates that the platform increases the standard quality, prompting substantial growth in loan volumes to avoid a collapse of the market. Except for the debt-to-income (DTI) ratio that positively impacts the response variable, the other hard information such as months since recent inquires is negative but significant predictors. It indicates that the Fintech platform focuses widely on the DTI in screening activity, neglecting additional credit risk information. In the second model, the previous misreporting variables are replaced from the two specifications of the roundness of the loan amount. The covariates are still strongly significant after controlling for other borrower information (e.g., home status and employment length). The roundness of loan amount varies between different thresholds, highlighting a decrease of 20.9% in the odds of being in the best category of prudence corresponding with the higher level of roundedness. The last model shows that the screening quality does not increase when some borrowers provide a longer description of the loan. However, borrowers who give a long loan purpose description are associated with high rates of default. This interpretation is consistent with the view that the platform’s screening quality is careless in distinguishing between good and bad loans. In contrast, the level of prudence is strengthened versus borrowers who report a length of employment at an extreme value, not confirming the hypothesis that the platform neglects borrowers who state a maximum period of work. The prescreening activity appears to be more severe versus borrowers who use loans to invest in small business purposes. At the same time, there is little prudence against the debt consolidation and credit card purposes that represent the majority of the loans issued on the platform.

### Robustness checks

To ensure the robustness of the regression results presented in the last section, I have adopted a new classification of the FICO score based on a further criterion. The new assessment of FICO scores is based on the percentiles taken from the variable. Specifically, the first cut off point (FICO <  = 667) takes all values below the 10th percentile; the second bin takes all values within the 10th and 25th percentiles (668–677); the third takes all values within the 25th and 50th percentiles (678–692); the fourth takes all values within the 50th and 75th percentiles (693–717); the fifth takes all values within the 75th and 90th percentiles (718–747); the sixth takes all values within the 90th and 95th percentiles (748–767); the last bin takes all values within the 95th and the 99th percentiles (FICO > 767). Table 5 shows the regression results performed by using as the response variable, the prudence grade constructed on the new assessment of FICO scores, with the same predictors as those shown in Table 4. As we can see, the regression results appear almost unchangeable by confirming the robustness of the previous results.

**Table 5:** Regression results with the dependent variable Prudence grade

	(1)		(2)		(3)	
	Low-Prundence	Medium	Low-Prundence	Medium	Low-Prundence	Medium
Not verified	0.875***	0.663***	0.719***	0.523***		
	(0.00441)	(0.00578)	(0.00352)	(0.00444)		
Income div. 5000	0.929***	0.908***				
	(0.00550)	(0.00852)				
Income div. 10,000	0.998	0.974**				
	(0.00648)	(0.0101)				
Amount div. 5000			1.128***	1.162***		
			(0.00681)	(0.0110)		
Amount div. 10,000			0.814***	0.745***		
			(0.00609)	(0.00892)		
Length of title					0.999	0.934***
					(0.00622)	(0.00904)
Suspect empl					1.052***	1.020**
					(0.00586)	(0.00895)
ln(Loan amount)	0.00168***	0.00166***			0.00309***	0.00248***
	(0.000111)	(0.000179)			(0.000248)	(0.000321)
ln(Loan amount)^2^	1.460***	1.468***			1.420***	1.449***
	(0.00519)	(0.00847)			(0.00614)	(0.0100)
Debt to income ratio	1.039***	1.044***	1.042***	1.044***	1.041***	1.046***
	(0.000279)	(0.000426)	(0.000287)	(0.000439)	(0.000345)	(0.000530)
Months since recent inq	0.988***	0.972***	0.990***	0.974***	0.989***	0.973***
	(0.000392)	(0.000631)	(0.000388)	(0.000626)	(0.000480)	(0.000767)
Revolving utilitation	0.0422***	0.0440***	0.0491***	0.0523***	0.0496***	0.0592***
	(0.000449)	(0.000676)	(0.000511)	(0.000794)	(0.000652)	(0.00112)
Account open	0.954***	0.967***	0.948***	0.963***	0.959***	0.977***
	(0.000712)	(0.00106)	(0.000707)	(0.00104)	(0.000872)	(0.00127)
Delinquencies in last 2y	0.622***	0.625***	0.607***	0.610***	0.616***	0.620***
	(0.00268)	(0.00403)	(0.00261)	(0.00389)	(0.00321)	(0.00479)
Purpose: credit card					0.328***	0.245***
					(0.00438)	(0.00533)
Purpose: debt consol					0.555***	0.485***
					(0.00488)	(0.00626)
Purpose: home					0.671***	0.585***
					(0.00835)	(0.0109)
Purpose: vacation					0.947*	0.950
					(0.0297)	(0.0451)
Purpose: small business					1.388***	1.464***
					(0.0319)	(0.0422)
Home mortgaged			1.221***	1.085***	1.085***	0.908***
			(0.00609)	(0.00859)	(0.00675)	(0.00886)
Home owned			1.175***	1.164***	1.113***	1.094***
			(0.00886)	(0.0135)	(0.0105)	(0.0157)
Year					0.794***	0.745***
					(0.00454)	(0.00672)
3-digit zip code					Yes	Yes
Observations	1,558,546	1,558,546	1,558,546	1,558,546	1,053,512	1,053,512

The dependent variable is created by comparing the LC grade and the new classification of FICO scores based on the percentiles. This table displays odds ratios from Gologit regressions that m-1 models, where m is the number of clusters. The dependent variable is an indicator of Prudence'screening by platforms, and the highest Prudence is the reference category. Prudence degree takes value 1 (Low Prudence) if LC rating grade has been underestimating borrowers'credit risk; Prudence degree takes value 2 (Medium) if the assessing of the borrower riskiness is the same in LC rating grade and FICO score; Prudence degree takes value 3 (High Prudence) if LC rating grade has been overestimating borrowers'credit risk.Imprudent models are displayed in columns 1, 3 and 5, and neutral groups in Columns 2,4 and 6. The variables with superscript α meet the odds assumptions and are the same in all categories (e.g. debt-to-income ratio, home mortgaged, home owned). For variables violating proportional odds assumptions refer to coefficients for responses of 2,3 vs 1 group (Low Prudence models) and category 3 vs 1, 2 in Neutral models

## Calculation of recovery rate

In the previous section, I have investigated the platform’s misconduct through the prudence index by confirming the Fintech platform’s inability to detect some misreporting borrowers. This section explores whether the lack of a verification process of the information reported by borrowers (e.g., annual income and employment length) harms the lenders’ collection performance.

To date, the LGD and RR are less studied than the probability of default in the P2P lending market. RR represents the proportion of money that lenders can successfully recover once the borrower has defaulted on the funding minus the administration fees during the collection period. In contrast, LGD is defined as the proportion of money investors fail to recover, given that the borrower has already defaulted. The equations of LGD and RR are reported below:

2 $$\mathrm{Recovery Rate}=\frac{\sum \mathrm{ Recoveries }- \sum \mathrm{ Collection recovery fee }}{\mathrm{Exposure at Default}}$$

and

3 $$\mathrm{Loss Given Default}=1- \frac{\sum \mathrm{ Recoveries }- \sum \mathrm{ Collection recovery fee }}{\mathrm{Exposure at Default}}$$

Typically, the RR and LGD lie in the interval (0,1) with high peaking values at the boundary levels 0 and 1. The RR could be less than 0 if recoveries are lower than the administration fee and greater than 1 if recoveries are more than the collection fee. The denominator is defined as the outstanding loan balance when the loan defaults. The RR of all default loans issued on LendingClub is estimated with Eq. (2), and LGD with Eq. (3). Variables are winsorized at 1% and 99% levels to mitigate the influence of outliers. The descriptive statistics of the RR and the LGD are listed in Table 6.

**Table 6:** Descriptive statistics of RR and LGD

Variable of interest	Number sample	Mean value	Median value	Standard deviation	Minimum value	Maximum value
Loss given default	291,664	0.902	0.867	0.109	0	1
Recovery rate	291,664	0.098	0.089	0.109	0	1

Notes: In this table, the chief summary statistics of RR and LGD are listed. Following literature in mortgage loans, the RR and LGD have truncated within the interval [0, 1]

**Fig. 7:**
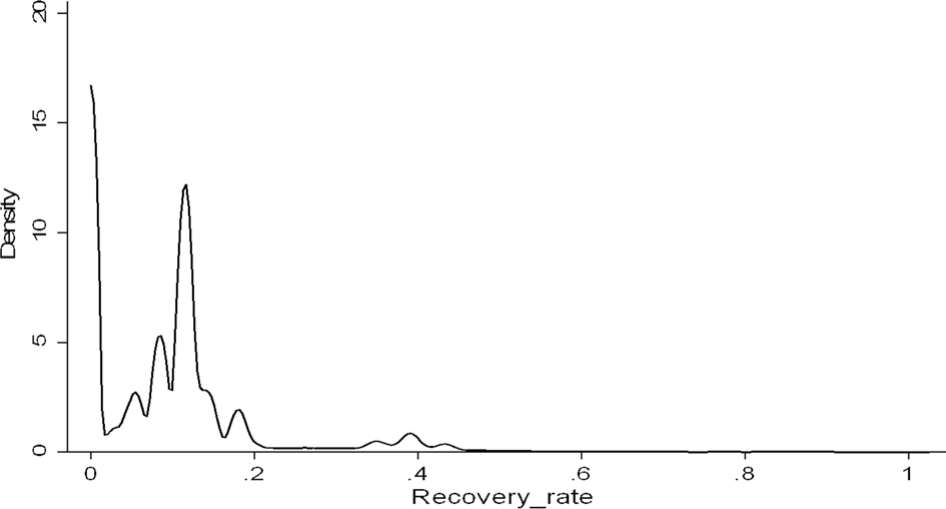
Kernel density of recovery rates in the sample. Notes. This graph shows the density distributions of the recovery rates on defaulted loans in the sample. The stack of 0 s shows the frequency of RR = 0, resulting in a not unimodal distribution

As shown above, the average values of the RR and LGD are 9.8% and 90.2%, respectively, indicating a sizeable total default loss and insufficient collection. It suggests that LendingClub originates loans with extreme credit risk, consistent with the lower RR value in the overall unsecured market. To test the normal assumptions of the empirical distribution of the RR, the density function is estimated using the kernel method of defaulted loans of LendingClub, resulting in the distribution displayed in Fig. 7. It can be seen clearly that the RR does not follow the normal distribution, with a high spike at boundary value 0 and several peaking values at 0.15. It is further strengthened by the Kolmogorov–Smirnov test that I applied as a robustness check. Analysis results of the RR and LGD of defaulted loans of LendingClub show that the priority for protecting lenders against credit risk is relatively low, suggesting that the Fintech platforms have carried out feeble efforts in the debt collection activity.

**Fig. 8:**
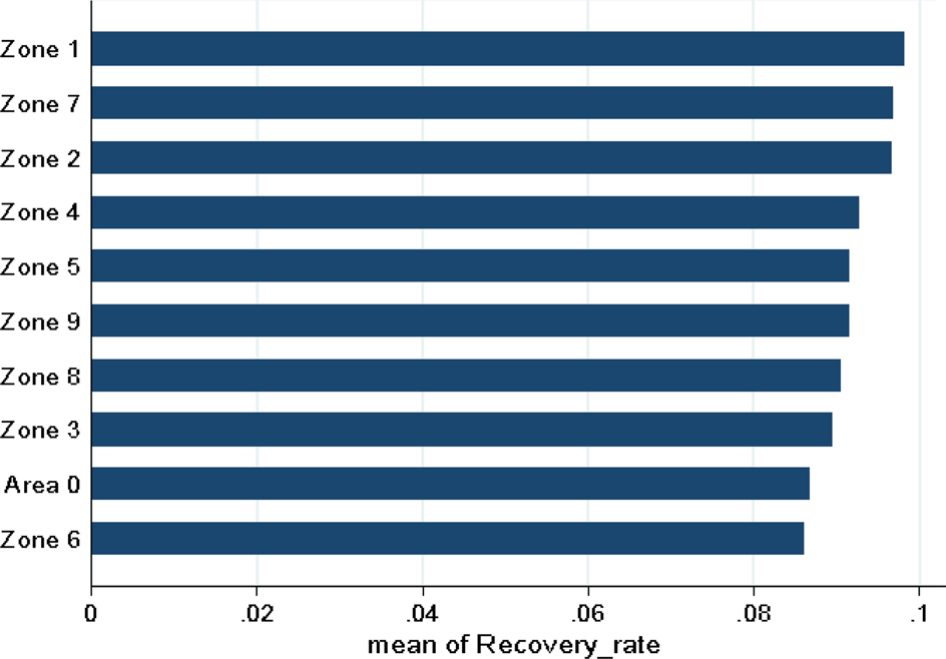
Mean-recovery rate by country. Notes. This graph shows the average distribution of the Recovery Rate of the loans issued on the LendingClub platform by each country. The US is classified in 10 zones based on the borrower’s first three digits of the ZIP code

Moreover, it is observed that the mean recovery rate is country-level heterogeneous, as presented in Fig. 8. The borrowers’ locations are based on the first three-digit ZIP code, captured into ten dummy variables concerning the classification of the United States. The lowest recovery rate is between zone 0 and zone 6 where, for instance, Connecticut, Massachusetts, Illinois, and other states are included.

The RR modeling has risen as a challenging task since it does not have a normal distribution. Recent statistic models have proposed a two-stage model; mixed continuous-discrete distributions.^17^ Beta regression, zero–one inflated beta regression, beta mixture models with logistic regression, and fractional regression have been applied, as shown in Table 7. The models’ prediction performances have been estimated through two indexes of accuracy, namely, root mean square error (RMSE) and mean absolute error (MAE).^18^ All models have been trained on the same dataset, avoiding potential bias due to different data. In applying the standard beta regression, I have adopted the transformation of the response variable proposed in the work of Smithson and Verkuilen (2006) to include 0 and 1 values. In terms of predictive power, the zero–one inflated beta regression model seems to perform better than others. Based on these findings, the RR is being modeled with the zero–one inflated beta regression.

**Table 7:** Models’ comparison

Model	RMSE	MAE
Standard beta regression	0.123	0.0050
Zero–one inflated beta regression	0.003	0.0003
Beta with logistic regression	0.005	0.0091
Fractional regression with logit estimator	0.129	0.0048

This table lists the predictive results of different approaches applied in the empirical analysis. The dependent variable is the recovery rate on defaulted loans issued from LendingClub between July 2007 and September 2018

### Zero-inflated beta regression of recovery rate

In the last sections, I focused on RRs’ density function for LendingClub loans. What are the determinants of the low RRs? Are they the same within each risk class? This section aims to present RR modeling on non-performing loans through a mixed continuous-discrete model adopted in the literature to estimate LGD in the unsecured market. I perform the zero–one inflated beta regression (ZOIB)^19^ with two components, which are simultaneously developed: (1) a logistic regression that models the predicted probability for whether or not borrowers have no recovery rate (RR = 0); and a (2) beta regression model that analyses the degree of RR between 0 and 1 (0 < RR < 1). Following Cragg (1971) and Cook et al. (2008), the logit link is used to model p_i_ as a function of explanatory variables, defined from the following equations:

4 $$\mathrm{Logit}\left({\mathrm{p}}_{\mathrm{i}}\right)={\mathrm{\alpha }+\upbeta }^{\mathrm{^{\prime}}}\mathrm{Notverified}+\updelta {\mathrm{Z}}_{\mathrm{i}}$$

The response variable is RR on non-performing loans in the sample. Not verified is an indicator variable that reflects the status of loan verification for the information submitted by applicants. The vector Z_i_ includes a set of controls, for instance, loan characteristics (interest rate, loan amount, and maturity), borrowers’ solvability information (debt to income ratio, months since the last delinquency, number of inquiries, number of banking accounts, rate of revolving utilization, mortgage account) and self-reported variables (annual income, borrower working years, homeownership status, loan purpose, state of borrower). The verification process is used as a second proxy for analyzing the platform’s misconduct, as the lower frequency of borrower verification could hide an attempt to boost loan volume. I regress the RRs of defaulted loans on the platform’s verification process, and the results are shown in Table 8.

**Table 8:** Regression results recovery rate in the overall sample

	(1)		(2)		(3)	
	Beta	Zero-inflate	Beta	Zero-inflate	Beta	Zero-inflate
Not verified	− 0.042*** (0.0043)	0.1169*** (0.0100)	− 0.0054*** (0.0058)	0.138*** (0.0146)	− 0.0412*** (0.0055)	0.0896*** (0.0130)
ln(Loan Amount)	0.0003 (0.0030)	0.0445*** (0.0738)	− 0.0049 (0.0057)	− 0.0531*** (0.0110)	0.0088 (0.0099)	− 0.0183 (0.0230)
Term	0.028*** (0.0042)	0.172*** (0.0102)	− 0.0061*** (0.0005)	0.171*** (0.0149)	− 0.00407*** (0.0055)	0.259*** (0.0135)
Interest rate	− 0.0006 (0.0004)	− 0.026 (0.0009**)**	− 0.024*** (0.0042)	− 0.0225*** (0.0014)	− 0.00126*** (0.0005)	− 0.0334*** (0.0012)
Revolving utilitation			− 0.0539*** (0.0139)	− 0.338*** (0.0364)		
Months since last delinquent			0.0006*** (0.0001)	0.00215*** (0.0003)		
Total account			0.00137*** (0.0002)	− 0.0015*** (0.0005)		
Bankcard balance > 75%			6.94e−05 (9.06e−05)	0.0009*** (0.00024)		
Debt to income ratio			0.00182*** (0.0002)	0.00850*** (0.0006)		
Mortgage account			− 0.0053*** (0.0013)	− 0.0070** (0.0035)		
ln(Annual Income)					0.0282*** (0.0106)	− 0.0423* (0.0245)
Employment length					0.00100 (0.0007)	0.00764*** (0.00165)
Loan to annual income					− 0.263*** (0.0482)	1.008*** (0.105)
Loan purpose: credit card					0.0264*** (0.0098)	0.135*** (0.0242)
Loan purpose: debt consolidation					0.0243*** (0.00870)	0.0775*** (0.0217)
Loan purpose: small business					− 0.146*** (0.0190)	− 0.0550 (0.0492)
Home mortgaged					− 0.00809 (0.103)	− 0.0492** (0.299)
Home owned					0.0432 (0.103)	− 0.0950* (0.299)
3-digit zip					Yes	Yes
Year					0.191*** (0.00458)	0.716*** (0.0112)
Observations	291,664		145,646		177,963	
AIC	− 105,632		− 62,897		− 73,825	
BIC	− 105,516		− 62,630		− 73,381	

The table reports results from beta regression and the logit model on RR with indicators and continuous explanatory variables. Both in the beta models and zero-inflated are reported the coefficients. The standards errors are in parentheses. All models are estimated with intercepts. The primary independent variable is associated with the verification process. Other control variables are inserted, like loan contract information and borrower’ characteristics. For brevity, only the loan’s significance is exposed. The borrowers’ state is based on the first three-digit ZIP code, captured into ten dummy variables building on the classification of the United States. The estimated goodness of fit is shown

***, ** and * denotes significance at levels 1%, 5%, and 10% levels, respectively

The dependent variable is the RR on defaulted loans, and it is the same in all models. The results are presented both through the beta regression (e.g., for proportional values, 0 < RR < 1) and logistic models (whether or not RR = 0). The first model investigates the relationship between RR and loan contract information and mainly reflects the variable Not Verified on the RRs. The first result of this model shows an increase in borrowers with incomplete verified information results in the lower RRs. Specifically, the coefficients for Not Verified are negative in the beta models, reflecting a decrease in RRs’ proportional value, and positive in the zero-inflated component by increasing the predicted probability that investors could not have recovered the rate after the borrowers were charged off (logit component). In terms of practical significance, the change of the variable from 1 to 0 decreases the response variables by 0.7 ppt, as shown in Table 9.

**Table 9:** Average marginal effects

	(1)		(2)		(3)	
	AME	SE	AME	SE	AME	SE
Not verified	− 0.00681***	0.000457	− 0.00403***	0.000645	− 0.00591***	0.000590
ln(Loan Amount)	0.00120***	0.000326	− 0.000854*	0.000482	0.00524***	0.00105
Term	− 0.00692***	0.000454	− 0.00505***	0.000650	− 0.00678***	0.000593
Interest rate	0.000653***	4.25e−05	0.000017	6.33e−05	0.000734***	5.57e−05
Revolving utilitation			0.00845***	0.001201		
Months since last delinquent			− 0.00009***	0.00001		
Debt to income ratio			− 0.00004*	0.00003		
Mortgage account			− 0.000302**	0.00001		
Bankcard Balance > 75%			− 0.000186***	0.00003		
Total account			0.00019***	0.00002		
ln(Annual Income)					0.00304***	0.00112
Loan to annual income					− 0.0495***	0.00501
Home mortgaged					− 0.0144	0.0118
Home owned					− 0.0142	0.0118
Home rented					− 0.00849	0.0118
Loan purpose: credit card					− 0.00149	0.00106
Loan purpose: debt consolidation					− 0.000265	0.000943
Loan purpose: small business					− 0.0111***	0.00209
Employment length					0.000146**	7.31e−05
Year					− 0.00107**	0.000493
Observations						
	291,664		146,246		177,963	

The table report results from beta regression and logit model on RR with indicators and continuous explanatory variables. Both in the beta models and zero-inflated are reported the average marginal effects. The standards errors are in paratheses. All models are estimated with intercepts. For brevity, only the loan purpose' significant are exposed. The estimated goodness of fit is shown

***, ** and * denotes significative at levels 1%, 5% and 10% levels, respectively

Regarding loan contract information, the signaling effect of the interest rate on borrower riskiness seems to fail. The negative coefficients of the interest rate in the beta specifications suggest that borrowers with higher interest rates do not present an RR equal to zero. This result sheds light on the pricing mechanism of loans applied for by the platform, resulting in the need to improve it. In the second specification, some control variables related to borrowers’ indebtedness have been added, and the effect of Not Verified on the RRs remains unchanged. Borrowers with banking accounts in which the balance is in the upper-to-high limit and whose last delinquency occurred recently cause a decrease of RR, as reported from the positive coefficient in the zero-inflated specifications. However, borrowers with repeated bank relationships, measured in terms of the total banking accounts and revolving utilization, significantly impact RR in the zero-inflated component. It might suggest that the tie between borrowers and banks could enhance their accountability to avoid losing reputation and raise future funding from banks. Finally, in the third model, variables related to the total assets of the borrower provided by the Credit Bureau have been dropped, and self-reported information has been added. Concerning these variables, regression results indicate that RR is significantly negatively correlated with the borrowers’ indebtedness ratio (e.g., loan amount to annual income). This finding implies that an excessive increase in debts beyond the safety threshold can absorb the majority of that income, undermining the borrower’s solvability. Housing ownership has a low significant impact on RR for the mortgaged borrower, unlike the borrower’s annual income, which causes a significant positive influence on RR.

Borrowers who decide to use the funding for small business development have lower RRs than those who apply for loans for credit card and debit consolidation purposes. The negative and significant relationship between the dummy year and RR confirms a decrease in RRs over the last years. It might be a potential consequence of fraud detection by the SEC at the beginning of 2016. The suspicion of being a dishonest broker could have decreased accountability by consumers and encouraged fraudulent behavior. The quality of the three specifications has been tested through the Akaike Information Criterion (AIC) and the Bayesian Information Criterion (BIC). The first model achieves the best goodness of fit for both parameters. Consequently, loan contract information seems to be the best predictor of RRs in our empirical analysis.

### Regression results of the recovery rate within risk classes

In the previous section, the zero–one inflated beta regression was applied to all risk classes based on the LC grade. This section aims to investigate the determinants of the RRs within each loan risk class separately. To achieve this goal, the risk classes are subdivided into low-risk class (involving loans with grade A or B), medium-risk class (loans with grade C or D), and high-risk class (loans with grade E–F or G). The zero–one inflated regressions for the three risk classes allow evaluation of the difference between the regression result from given risk classes and the whole dataset. Regression results within each risk class are displayed in Table 10.

**Table 10:** Regression results within each risk-class

	(1) Low-risk class		(2) Medium-risk class		(3) High-risk class	
	Beta	Zero-inflate	Beta	Zero-inflate	Beta	Zero-inflate
Not verified	− 0.0415*** (0.0116)	0.0762*** (0.0264)	− 0.0298*** (0.0061)	0.128*** (0.0151)	− 0.0137*** (0.0106)	0.136*** (0.0273)
ln(Loan Amount)	0.0369 (0.0242)	− 0.170*** (0.0533)	0.00493 (0.0109)	− 0.185*** (0.0269)	− 0.00356 (0.0176)	− 0.223*** (0.0455)
Term	− 0.0144 (0.0181)	0.462*** (0.0383)	− 0.0225*** (0.0060)	0.140*** (0.0155)	− 0.0146* (0.0086)	0.0212 (0.0234)
Interest rate	− 0.0529*** (0.00314)	− 0.122*** (0.00733)	0.0033*** (0.0006)	0.0233*** (0.0015)	0.00233* (0.0012)	0.0251*** (0.0031)
Revolving utilitation	0.0098 (0.0245)	− 0.589*** (0.0572)	− 0.0829*** (0.0109)	− 0.449*** (0.0281)	− 0.108*** (0.0164)	− 0.310*** (0.0444)
Months since last delinquent	0.0008*** (0.0002)	0.0024*** (0.0005)	0.0005*** (0.0001)	0.0019*** (0.00029)	− 0.00026 (0.0002)	0.00150*** (0.0004)
Debt to income ratio	0.00629*** (0.0007)	0.0096*** (0.0016)	0.00502*** (0.0003)	0.0079*** (0.0007)	0.0051*** (0.0004)	0.0071*** (0.0011)
ln(Annual Income)	0.00741 (0.0251)	0.0871 (0.0561)	0.0399*** (0.0115)	0.0778*** (0.0288)	0.0121 (0.0187)	0.0105 (0.0488)
Employment length	0.00286* (0.00160)	0.00256 (0.00372)	0.0033*** (0.0007)	0.0029 (0.0019)	0.003*** (0.0011)	0.00212 (0.00303)
Loan to annual income	− 0.660*** (0.142)	1.362*** (0.295)	− 0.250*** (0.0543)	1.039*** (0.130)	− 0.204** (0.0799)	0.945*** (0.201)
Loan purpose: credit card	0.0601*** (0.0230)	− 0.0692 (0.0514)	0.00690 (0.0103)	0.0758*** (0.0263)	− 0.00121 (0.0162)	0.142*** (0.0436)
Loan purpose: debt consolidation	0.0370* (0.0215)	− 0.104** (0.0477)	0.00038 (0.00868)	0.0267 (0.0225)	0.00493 (0.0124)	0.116*** (0.0338)
Loan purpose: small business	− 0.170*** (0.0575)	− 0.427*** (0.142)	− 0.106*** (0.0201)	− 0.105* (0.0545)	0.069*** (0.0261)	− 0.187** (0.0769)
Home mortgaged	− 0.506* (0.282)	− 0.579 (0.635)	0.0352 (0.136)	0.142 (0.363)	0.387* (0.198)	0.397 (0.559)
Home owned	− 0.427 (0.283)	− 0.610 (0.636)	0.0682 (0.136)	0.0772 (0.363)	0.420** (0.198)	0.293 (0.560)
3-digit zip						
	Yes	Yes	Yes	Yes	Yes	Yes
Constant	− 1.159*** (0.323)	0.494 (0.731)	− 2.281*** (0.751)	− 0.774* (0.406)	− 2.394*** (0.2271)	− 0.757 (0.635)
Observations	32,906		136,791		56,652	

The dependent variable is RR within each risk class. In both models, estimated coefficients are reported. Standard errors are in parentheses

*, ** and *** denotes 1%, 5% and 10% significative levels, respective

The results, as shown in Table 10, provide additional support for the results in the last section. The indicator variable related to the verification process of borrower data decreases the average proportion of RR within each risk class, confirming the previous findings in the whole dataset. Regarding bank transactions borrower variables, the regression results for each class separately and the full dataset only differ slightly. For the loan contract information, the variable loan amount is a significant negative predictor in zero-inflation models in all risk classes, suggesting that an increase of the amount causes the decrease of predicted probability (RR = 0) with a significant effect in the low-risk class. The coefficients term of the loan, months since the last delinquency, and loan amount to annual income ratio are negative and highly significant in all risk classes. The variable interest rate is a negative predictor in low-risk and high-risk classes, but it is not significant in the medium-risk class. The interest rate should be an essential predictor of the borrower’s riskiness. However, it yields a correct prediction only in the low-risk category, reducing the RR on average by 0.002 points. Concerning self-reported information, the borrower’s annual income is not significant in the low-risk and high-risk classes, while it is significant in the medium-risk class. The length of employment positively impacts the RR in the medium- and high-risk categories but not in the low-risk ones. The strong negative relationship between small business purpose and RR is confirmed only in the medium-risk class. Overall, the robustness test demonstrates that the main predictor of the RRs is the verification status of the loans. Strengthening the verification process might lead to advantages both for lenders in mitigating the harmful effects of the LGD and for the same platform in terms of reputation. Moreover, these results underline the weakness of the risk management framework adopted by the LC, as the low RR could highlight the careless screening activity of borrowers (Table 11).

**Table 11:** Average marginal effects

	(1) Low-risk class		(2) Medium-risk class		(3) High-risk class	
	AME	SE	AME	SE	AME	SE
Not verified	− 0.00600***	0.00129	− 0.00597***	0.000671	− 0.00449***	0.00115
ln(Loan Amount)	0.00923***	0.00189	0.00853***	0.000843	0.00622***	0.00133
Term	− 0.0155***	0.00197	− 0.00572***	0.000672	− 0.00188**	0.000958
Interest rate	− 0.00107***	0.000354	0.000282***	6.63e−05	− 0.000415***	0.000132
Revolving utilitation	0.0195***	0.00276	0.00509***	0.00121	− 0.00165	0.00181
Months since last delinquent	− 0.00016***	2.77e−05	− 0.000105***	1.26e−05	− 6.75e−05***	1.90e−05
Debt to income ratio	0.000307***	7.78e−05	0.000267***	3.24e−05	0.000304***	4.70e−05
ln(Annual Income)	− 0.00217	0.00166	− 0.00174**	0.000737	− 5.18e−05	0.00117
Employment length	0.000236	0.000181	0.000258***	8.24e−05	0.000253**	0.000125
Loan to annual income	− 0.101***	0.0108	− 0.0633***	0.00420	− 0.0447***	0.00604
Loan purpose: credit card	0.00879***	0.00249	− 0.00129	0.00112	− 0.00381**	0.00178
Loan purpose: debt consolidation	0.00775***	0.00232	− 0.000549	0.000952	− 0.00260*	0.00136
Loan purpose: small business	− 0.00140	0.00663	− 0.00683***	0.00227	− 0.00226	0.00297
Home mortgaged	− 0.0277	0.0314	0.00167	0.0153	0.0272	0.0222
Home owned	− 0.0198	0.0315	0.00626	0.0153	0.0327	0.0222
Observations	32,906		136,791		56,652	

This table reports the average marginal effects of the models presented in Panel A. The dependent variable is RR within each risk class. Standard errors in parentheses

*, ** and *** denote 1%, 5% and 10% levels of significance, respectively

## Discussion and conclusion

P2P Fintech platforms represent an essential source of alternative funding, thereby fostering credit democratization. Lending platforms perform functions similar to that of traditional intermediaries, such as loan evaluation, pricing, and screening activity (Balyuk and Davydenko 2019). However, lending platforms have a challenging position because of the trade-off between improving the borrower screening activity and maximizing loan volume. Their incentives could lead them to boost loan originations by decreasing credit quality. This paper presents some significant findings regarding how players’ incentives shape their behavior and the theoretical and practical implications, as discussed below. First, this study contributes to the literature on financial misconduct and P2P lending by exploring the impact of platforms’ incentives on assessing borrower riskiness. Although a growing body of research provides valuable discussions of the determinants of default and funding success in the crowdfunding market (e.g., Lee and Lee 2012; Morse 2015; Serrano-Cinca et al. 2015), how the platforms’ incentives affect their behavior in terms of the borrower screening activity has not yet been investigated. My regression results show that the degree of prudence taken by the lending platform does not improve whether some borrowers report misleading information. However, I find that the platform increases the standard of quality only when some borrowers present misreporting characteristics that are easier to identify, such as the self-reported length of employment at an extreme level. The screening quality of the LC marketplace seems to have decreased over the past years.

On the one hand, it could be ascribable to challenging times in the sector and the episodes of fraud detection in general. On the other hand, the financial restatements imposed by SEC and DOJ may have prompted LendingClub to adopt more aggressive loan underwriting, thereby resulting in a decrease in prudence. I also show that the borrowers’ rating does not increase when the platform does not verify some loans. According to the signaling theory in the crowdfunding market (Ahlers et al. 2015), whether the Fintech platforms would adopt due diligence at loan origination, such as strengthening the verification process for information self-reported by borrowers, could signal their trustworthiness in the market. Second, this work contributes to the P2P lending literature by exploring the relationship between the determinants of recovery rate and the lack of verification process on the information reported by borrowers. Few studies have analyzed the key drivers of the recovery rate in the P2P lending market (Pursiainen 2020; Zhou et al. 2018). The regression results suggest that borrowers with incompletely verified information negatively affect the recovery rate, harming the lenders’ collection performance. Although this paper is focused only on one P2P lending platform, and as such, the external validity of the findings may not be generalizable, it provides some preliminary practical insights for lenders, consumers, and policymakers to guarantee the market’s survival.

This research has important implications for lenders in the P2P lending market. The results suggest that the lending platform is unable to detect some misreporting borrowers, thus harming the lenders’ collection performance. My results are consistent with the theoretical insights from behavioral finance and psychology (Chao 2021; Jansen and Pollmann 2001), stating that misreporting borrowers have a higher tendency to communicate round values about their assets. Thus, these results can help lenders in marketplace lending make more informed investment decisions by incorporating an index of borrowers’ misreporting (e.g., the rounding of income, the rounding of amount) into their decision-making process. Also, this research encourages lenders to be mindful of the default rates and the LGD in assessing the credit risk of loans. This research provides additional knowledge regarding the dynamics of the crowdfunding market, thereby enhancing understanding of the platform’s role in the governance of lending marketplaces. Policymakers should pay attention to this market as lending platforms act without skin in the game, do not take deposits, and do not perform maturity transformation. This would make them vulnerable to decreased standards in loan evaluations. Thereby, policymakers should adopt due diligence to ensure that lending platforms constantly fulfill the prescreening activity’s standard quality.

The limitations of this study be overcome in further research. Future works might corroborate the findings using a new sample to increase external validity. For instance, the hypothesis developed in this work can be replicated in other crowdfunding platforms by comparing the different crowdfunding mechanisms (e.g., reward-based, donation-based, lending-based, and equity-based). These further studies can help understanding which typology of crowdfunding presents a higher likelihood of misconduct and whether the verification process works better depending on the model implemented. A second direction for future studies could include other factors accounted for in my studies, such as macroeconomics and cultural context, to explore whether the main results continue to hold in a different setting. Third, future research should investigate whether a cut-off point exists beyond which maximizing platforms’ incentives harms lenders. Finally, the socio-economic crisis due to the COVID-19 pandemic has been reshaping all economic activities. Researchers should investigate the crowdfunding market’s resilience in such an uncertain time and whether the standard quality in the screening of loans has decreased to restore the volume of loans.

## Data Availability

Data used in this paper were collected from LendingClub.

## Appendix: Further analysis

See Tables 12 and 13.

**Table 12:** Endogeneity check

	(1)	(2)	(3)
Verification process	0.0352*** (0.00493)	0.0554*** (0.00703)	0.0554*** (0.00703)
Rating grade	0.178*** (0.00541)	0.198*** (0.00783)	0.198*** (0.00783)
Term	0.485*** (0.00526)	0.545*** (0.00754)	0.545*** (0.00754)
Interest rate	6.503*** (0.144)	4.642*** (0.205)	4.642*** (0.205)
Revolving utilisation		− 0.111*** (0.0139)	− 0.111*** (0.0139)
Months since last delinquent		− 0.00138*** (0.000146)	− 0.00138*** (0.000146)
Total account		0.000630** (0.000294)	0.000630** (0.000294)
Debt to income ratio (DTI)		0.0163*** (0.000400)	0.0163*** (0.000400)
Mortgage account		− 0.109*** (0.00184)	− 0.109*** (0.00184)
ln(annual income)	− 0.238*** (0.00453)		
Constant	− 0.859*** (0.0504)	− 3.389*** (0.0170)	− 3.389*** (0.0170)
Observations	1,366,684	668,633	668,633

This table reports the results of logistic regression. The dependent variable is the probability of default in all columns. Standard errors in parentheses

*, ** and *** denote 1%, 5% and 10% levels of significant, respectively

**Table 13:** Endogeneity check: regressions for the three risk classes

	(1) A-B	(2) C-D	(3) E-F-G
Verification process	0.0408*** (0.0123)	0.0353** (0.0141)	0.0394*** (0.00796)
Term	0.601*** (0.0148)	0.549*** (0.0167)	0.532*** (0.00814)
Interest rate	18.60*** (0.271)	3.236*** (0.269)	7.754*** (0.166)
ln (annual income)	− 0.140*** (0.0103)	− 0.240*** (0.0147)	− 0.164*** (0.00776)
Loan purpose: debt consolidation	0.0391** (0.0175)	0.120*** (0.0204)	0.108*** (0.0116)
Loan purpose: credit card	0.0493*** (0.0188)	0.0514* (0.0286)	0.102*** (0.0139)
Loan purpose: home improvement	0.0822*** (0.0255)	0.0596* (0.0347)	0.118*** (0.0190)
Loan purpose: small business	0.662*** (0.0524)	0.283*** (0.0452)	0.370*** (0.0333)
Home mortaged	− 0.340*** (0.0113)	− 0.349*** (0.0155)	− 0.345*** (0.00839)
Home owned	− 0.127*** (0.0173)	− 0.215*** (0.0241)	− 0.197*** (0.0129)
year	0.350*** (0.0103)	0.193*** (0.0190)	0.272*** (0.00762)
3-digit zip	Yes	Yes	Yes
Constant	− 3.078*** (0.120)	0.579*** (0.167)	− 1.276*** (0.0887)
Observations	429,448	87,932	394,827

This table reports the results of logistic regression for all risk-classes. The dependent variable is the probability of default in all columns. In the first column the regression results for the lowest risk-class (A-B), in the second column the medium risk-class (C-D) and in the third column the highest risk-class (E-F-G) are shown. Standard errors in parentheses

*, ** and *** denote 1%, 5% and 10% levels of significant, respectively

## Footnotes

1 The term “Fintech marketplace” refers to the involvement of institutional investors within the P2P lending market in funding loans. For a detailed definition see FSB, 2017.

2 In December 2015, the Chinese Government accused Ezubao, an online lending platform, revealing that approximately 95% of its investment projects were fake and fabricated with information bought from other companies. The executive company confirmed the fraudulent activity and 21 Ezubao officials were arrested (Albercht et al. 2017).

3 Further, the investigation proved that a fund traced to the CEO who resigned had bought 115 million worth of LendingClub loans without previously disclosing the conflict of interest (DOJ settlements 2016).

4 LendingClub is already implicated in class-action lawsuits in California, where the company has been accused of “making materially false and misleading statements in the registration and prospectus issued with the IPO,” and in New York, where people received usury loans through the platform (Business Insiders 2016).

5 The boundaries value is modelled through the binary logistic regression (e.g., the predicted probability of RR being 0 versus not 0) and the continuous part (0–1) from the beta distribution.

6 In general, they argue that managers’ compensation ties to stock options provide them with several incentives in the engagement of aggressive accounting policies, which, in turn, results in improper conduct. Research on this topic is mixed, and there is still no predominant picture. For example, Erickson, Hanlon, and Maydew (2006) measured equity incentives of firms accused of fraud from the SEC, but they did not find a link beteen executive equity incentives and fraud. Findings of Armstrong et al. (2010) suggest that financial misstatement affects the trade-off risk-rewards, involving positive and negative effects, and equity incentives make it more likely when managers are less averse to equity risk. Burns and Kedia (2006) find that other components of compensation (i.e., salary plus bonus, equity, restricted stock) do not affect the propensity to misreport, as they do not introduce convexity in CEO wealth.

7 For instance, more than 70% of financial misconduct occurs in founder’s firms due to their overconfidence and hubris (Amiram et al. 2017).

8 Firms accused of financial misreporting may be subject to direct costs as represented by monetary fines and other penalties that have the potential to reduce approximately 38% of the firms’ value (Karpoff et al. 2008).

9 Other studies have investigated the role of text descriptions for predicting loan default through text mining analysis (see Herzenstein et al. 2011; Gao and Lin 2012; Nowak et al. 2018) and the likelihood of discrimination against black or female borrowers (see Pope and Sydnor 2011; Duarte et al. 2012; Ravina 2012; Loureiro and Gonzalez  2015, Dorfleitner et al.  2016).

10 Using a dataset from LendingClub, they state that more significant variables of default are credit grade assigned by LC and involve credit line utilization. For instance, Polena and Regner (2018), using a LendingClub dataset from January 2009 to December 2012, found that annual income, credit grade, inquires in the past six months, loan purpose, credit card, and small business are significant determinants of default within each risk class.

11 Large strands of literature focus on the moderator effects of financial misconduct (Li et al. 2017, Nguyen et al. 2015) and explore the role of skin in the game in discouraging any wrongdoing (Gorton and Pennacchi 1995; Holmstrom and Tirole 1997).

12 Estrada and Zamora (2016) state that a lower screening cost and a higher benefit from projects act as incentives to screen carefully.

13 In early 2009, LendingClub started a new program “Test proposal.” In Proposal T1, the platform approved loan applications from borrowers who did not meet certain requirements. Initially, these loans were declined by the Credit Department, but they were successfully approved under the TP1 program. In February 2009, LendingClub began TP2 programs by approving loans that did not overcome constraints as well as in TP1. The Fintech platform aimed to boost the loan origination volume. The Credit Department was aware of the underperformance of these, resulting in a punishment by the Department of Justice and the SEC (DOJ Settlement Agreements 2016).

14 It represents a performance measurement of a binary classifier by plotting the true positive rate (TPR) against the false positive rate (FPR). AUC (area under curves) measures the accuracy of the screening system, ranging from 0 (the worst tool) to 1 (perfect tool). Iyer et al. (2016) state that an AUC of 0.6 or greater is considered valuable in large information-asymmetry environments, while an AUC of 0.7 or greater is a desirable goal in rich-information contexts.

15 In specific: value 1 if FICO score is lower than 660; value 2, 3, 4, 5, 6 and 7 if it is 660 to 679, 680 to 699, 700 to 739, 740 to 759, 760 to 779, and above 780, respectively.

16 The largest odds ratio is identified in the neutral category that compares groups 3 vs 1 and 2. However, the effect is smaller when the income is rounded at a higher threshold.

17 This family of distributions, introduced by Ospina and Ferrari (2012), allows us to model data that assume values in [0, 1), (0, 1] or [0, 1].

18 Lower values of RMSE and MAE indicate a better fit, suggesting that the model can predict the response variable accurately.

19 Zero adjusted beta regression is more appropriate for modeling dependent variables containing large numbers of 0.
